# Orally delivered MK-4482 inhibits SARS-CoV-2 replication in the Syrian hamster model

**DOI:** 10.21203/rs.3.rs-86289/v1

**Published:** 2020-10-08

**Authors:** Kyle Rosenke, Frederick Hansen, Benjamin Schwarz, Friederike Feldmann, Elaine Haddock, Rebecca Rosenke, Kimberly Meade-White, Atsushi Okumura, Shanna Leventhal, David W. Hawman, Emily Ricotta, Catharine M. Bosio, Greg Saturday, Heinz Feldmann, Michael A. Jarvis

**Affiliations:** 1 Laboratory of Virology, Division of Intramural Research, National Institute of Allergy and Infectious Diseases, National Institutes of Health, Hamilton, MT, USA;; 2 Laboratory of Bacteriology, Division of Intramural Research, National Institute of Allergy and Infectious Diseases, National Institutes of Health, Hamilton, MT, USA;; 3 Rocky Mountain Veterinary Branch, Division of Intramural Research, National Institute of Allergy and Infectious Diseases, National Institutes of Health, Hamilton, MT, USA;; 4 Laboratory of Clinical Immunology and Microbiology, Division of Intramural Research, National Institute of Allergy and Infectious Diseases, National Institute of Health; Bethesda, MD, USA; 5 University of Plymouth, Plymouth, Devon, UK;; 6 The Vaccine Group Ltd, Plymouth, Devon, UK;

## Abstract

The COVID-19 pandemic progresses unabated in many regions of the world. An effective antiviral against SARS-CoV-2 that could be administered orally for use following high-risk exposure would be of substantial benefit in controlling the COVID-19 pandemic. Herein, we show that MK-4482, an orally administered nucleoside analog, inhibits SARS-CoV-2 replication in the Syrian hamster model. The inhibitory effect of MK-4482 on SARS-CoV-2 replication was observed in animals when the drug was administered either beginning 12 hours before or 12 hours following infection in a high-risk exposure model. These data support the potential utility of MK-4482 to control SARS-CoV-2 infection in humans following high-risk exposure as well as for treatment of COVID-19 patients.

## One sentence summary

MK-4482 inhibits SARS-CoV-2 replication in Syrian hamster model

## INTRODUCTION

Severe acute respiratory syndrome coronavirus 2 (SARS-CoV-2) is the causative agent of coronavirus disease 2019 (COVID-19)^1^. Following emergence of the virus in Wuhan in the Hubei province of the People’s Republic of China in late 2019^2^, COVID-19 was declared a pandemic by the World Health Organization (WHO) on 11^th^ March, 2020^3^. As of late September, 2020, there are over 32 million confirmed cases and more than 1,000,000 deaths from COVID-19 worldwide^3^. Myriad differences in governmental public health responses, politicization of the pandemic response and societal acceptance of control measures have resulted in differing levels of success in controlling the initial wave of infection around the world^4–7^. Even in those countries that have achieved a higher degree of control of the initial pandemic wave, the unavoidable need to relax highly stringent public health measures has resulted in a rebound of SARS-CoV-2 infections, with the associated fear of a second wave arriving this winter in the Northern hemisphere^8^.

Currently, there are no drugs suitable for high-risk exposure use against SARS-CoV-2. The nucleoside analog, GS-5734 (remdesivir), a non-obligate RNA chain terminator, has been granted emergency use authorization (EUA) by the FDA for the treatment of COVID-19 patients^9^. This EUA was based on the demonstration of a decreased time to recovery in patients hospitalized for severe COVID-19, and was recently expanded to include all hospitalized adult and pediatric patients, irrespective of disease severity^9,10^. In preclinical animal studies, which are more amenable than clinical trials for assessment against high-risk exposure, GS-5734 administered 12 hours after SARS-CoV-2 infection was shown to lower lung viral load and lung pathology, although treatment had no effect on shedding from the upper respiratory tract^11^. However, currently GS-5734 can be administered only via the intravenous route, which makes its application to the control of high-risk exposure challenging.

MK-4482 (known previously as EIDD-2801) is an orally administered bioavailable prodrug (5’-isopropylester form) of the cytidine nucleoside analogue EIDD-1931 (β-D-N^4^-hydroxycytidine; NHC)^12^. Using a high throughput screen of nucleoside analogs, EIDD-1931, the active compound resulting from hydrolysis of MK-4482, was identified as a broad activity inhibitor of influenza A and respiratory syncytial viruses, with initial functional assays showing the drug to function as a RNA mutagen rather than chain terminator^13^. Originally developed for treatment of hepatitis C virus (HCV) in early the 2000s^14^, recent studies indicated potent activity of EIDD-1931 against SARS-CoV-2 in multiple cell types, including biologically relevant epithelial cells *in vitro*, and against MERS-CoV-1 and SARS-CoV-1 coronaviruses in mouse models when administered shortly before as well as following infection^15^.

In the present study, we determined the half-maximal inhibitory concentration (IC_50_) value for EIDD-1931 in tissue culture and subsequently assessed the potential of MK-4482 following oral administration to control SARS-CoV-2 in the highly susceptible Syrian hamster model^16,17^. We show that MK-4482, when administered either starting at 12 hours prior to SARS-CoV-2 infection, or even 12 hours post-infection, significantly decreased viral lung loads and pathology, but did not affect shedding from the upper respiratory tract. These findings support the potential of MK-4482 as an orally administered drug for high-risk exposure and possibly therapeutic use in humans.

## RESULTS

**Figure 1:**
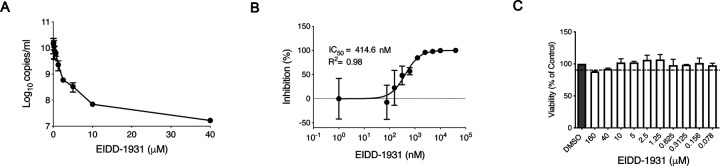
EIDD-1931 inhibits SARS-CoV-2 replication in human lung epithelial Calu-3 cells. Cells were pretreated for 1 hour with differing EIDD-1931 concentrations, followed by infection with SARS-CoV-2 at a MOI of 0.01 for 1 hour. After 1 hour, media was replaced, and cells were cultured in the presence of drug for 24 hours at 37°C in a 5% CO2 incubator. (**A**) Virus yield in the cell supernatant was measured by quantitative RT-PCR of clarified culture supernatant by using primer and probe sets to quantify total viral RNA (N gene; genomic and subgenomic RNA). (**B**) IC_50_ values were determined using results from the RT-PCR following log-based transformation of drug concentrations and normalization to percentage inhibition based on diluent alone controls by fitting to drug-dose response curves using Prism software. (**C**) Absence of toxicity (>90% viability; shown by dotted line) at highest EIDD-1931 concentration used for analysis of SARS-CoV-2 replication (40μM) was confirmed using CellTiter-Glo® 2.0 Assay (Promega, Corp., Madison, WI, USA) as per manufacturer’s protocol. For A to C, means are shown ± standard deviation.

First, we determined the *in vitro* inhibitory effect of EIDD-1931 on SARS-CoV-2 replication in Calu-3 cells, a disease-relevant human lung epithelial cell line. Cells were pretreated with differing drug concentrations and the effect on viral RNA load in tissue culture supernatant was determined at 24 hours after infection by quantitative reverse transcriptase polymerase chain reaction (RT-PCR) (Figure 1A). EIDD-1931 treatment resulted in a decrease in SARS-CoV-2 replication by approximately 3-logs (880-fold) when compared to no drug controls (Figure 1A). Viability was also assessed over the differing concentrations, demonstrating only minimal cellular toxicity at the highest drug concentration (Figure 1C). The half-maximal inhibitory concentration (IC_50_) value for EIDD-1931 was shown to be at sub-micromolar levels in Calu-3 cells at 414.6 nM (Figure 1B).

**Figure 2:**
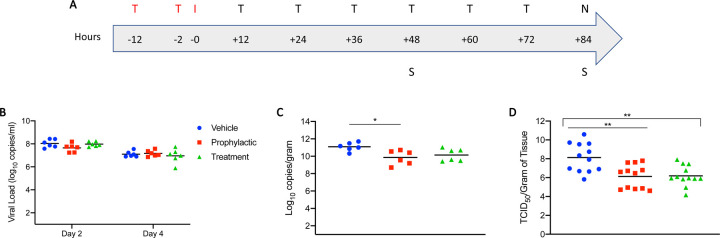
Syrian hamster model - Study design, viral shedding, viral load, infectious titers and viral antigen. (**A**) Study design. Hamsters were infected with SARS-CoV-2 by the intranasal route. MK-4482 was administered either pre-infection at 12 and 2 hours prior to infection, or post-infection with treatment started 12 hours post-infection. Treatment was then continued in both treatment groups every 12 hours for 3 consecutive days until end of the experiment. Animals were euthanized on day 4 and lungs were harvested for pathology and virology. ‘T’ denotes treatment (red: pre-infection and **black**: post-infection treatments); ‘I’ denotes infection; ‘S’ denotes swab samples and ‘N’ indicates necropsy. (**B**) Viral shedding. Oral swabs were collected on days 2 and 4 post-infection to measure viral shedding, determined by RT-PCR (N gene: genomic and subgenomic) (**C**) Viral load in lung tissue. Lung viral loads based on RT-PCR (N gene: genomic and subgenomic) were determined as a correlate for lower respiratory tract infection. (**D**) Infectious virus in lung tissue. Lung samples were homogenized and titered for infectious on Vero E6 cells. Infectious titers were determined as TCID_50_ equivalents using the Reed-Muench method^36^. Two independent lung samples were measured from each animal. (**B-D**) Blue circle, vehicle control; red square, pre-infection treatment; green triangle, post-infection treatment. **Summary of Results:** (**B**) No statistical significance in virus shedding was found between either of the two MK-4482 treatment groups and vehicle controls. (**C**) There was a significant difference in lung viral loads between the pre-infection group compared to the vehicle control. Although the post-infection group trended towards lower levels, there was no significant difference between this group and vehicle control. (**D**) Infectious titers in the lungs were significantly different between both pre-infection and post-infection groups, compared to vehicle control group, but no significance was found between treatment groups from each other. For B to D geometric means are shown. ANOVA followed by Kruskal-Wallis analysis and a pairwise Wilcox test was used to analyze differences among groups. *p<0.05, **p<0.008

Having verified *in vitro* efficacy and determined the IC_50_ value of EIDD-1931, we next assessed efficacy of the MK-4432 prodrug in the Syrian hamster model, which is regarded as a preclinical model of mild disease, with animals having self-limiting pneumonia^16,17^. Given the possibility for oral dosing, we were interested in the utility of MK-4432 as a treatment following high-risk exposure. Two groups of hamsters (n=6 per group) were treated with MK-4432 (250mg/kg) by oral gavage 12 hours and 2 hours before (pre-infection group) or 12 hours post-infection (post-infection group). Animals were then dosed every 12 hours with MK-4432 (250mg/kg). A control group was treated using the same route and timing as the pre-infection group with vehicle only (see schematic; Figure 2A). Hamsters were infected intranasally with SARS-CoV-2 using a dose of 5×10^2^ TCID_50_ (100 times infectious dose 50; ID_50_). The ID_50_ value was determined in a separate study concerned with further refinement of the Syrian hamster SARS-CoV-2 model^17^. Treatment in all groups was continued for 3 consecutive days and hamsters in all groups were euthanized on day 4 post-infection.

Disease manifestation in Syrian hamsters following SARS-CoV-2 infection is transient with only mild clinical signs^16,17^, and no discernible difference in disease manifestation based on clinical symptoms was observed between any group over the course of the study. Virus shedding was measured with oral swabs collected on day 2 and 4 post-infection. Levels of viral RNA in the oral cavity were similar between all groups at these two time points of analysis (approx. 10^8^ and 10^7^, for day 2 and 4 post-infection, respectively), and decreased from day 2 to 4 (Figure 2B). Lung tissue samples were collected at day 4 post-infection for analysis. In contrast to levels of shedding, a 1-log decrease in viral RNA was detected in the lungs of pre-infection and post-infection groups, respectively, when compared to the vehicle control group (Figure 2C). This corresponded to a 2-log decrease in infectious virus in the lungs of the MK-4482 treated groups when compared to the vehicle controls (Figure 2D).

**Figure 3:**
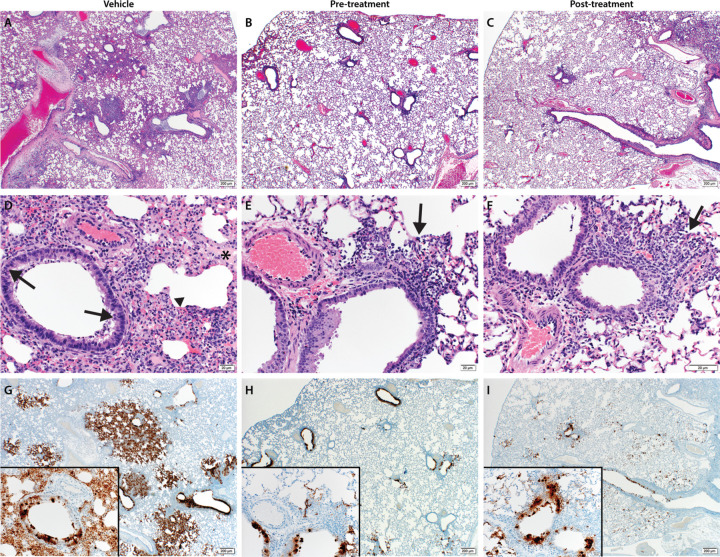
Pathological analysis of the lung tissue. Hematoxylin and eosin (H&E) staining was used on lung sections to examine lung pathology post-inoculation. Immunohistochemistry (IHC) was used to detect viral antigen in the same lung sections. (**A, D and G**) untreated vehicle control, (**B, E and H**) pre-infection treatment with antiviral drug MK-4482 and (**C, F** and **I**) post-infection treatment with MK-4482. (**A-F**) H&E stain (**G, H and I**) IHC for SARS-CoV-2 nucleocapsid antibody. (**A**) Lung 20X: multifocal, moderate broncho-interstitial pneumonia. (**B and C**) Lung 20X: minimal peribronchial interstitial pneumonia. (**D**) Lung 200X epithelial cell necrosis (**arrow**), edema (**asterisk**), interstitial pneumonia (**arrowhead**). (**E and F**) peribronchial and interstitial infiltrates (arrow). (**G**) Lung 20X; insert 200X: numerous immunoreactive bronchiolar epithelial cells, type I and II pneumocytes and fewer macrophages. (**H and I**) Lung 20X; insert 200X: scattered to moderate numbers of immunoreactive bronchiolar epithelial cells, type I and II pneumocytes and macrophages.

Lung samples were taken for histopathological analyses, and results are shown in Figure 3A to F. Analysis revealed pulmonary lesions consisting of a moderate-marked broncho-interstitial pneumonia centered on terminal bronchioles and extending into the adjacent alveoli. Multifocal necrotic epithelial cells and moderate numbers of infiltrating neutrophils and macrophages with abundant luminal cellular exudate in the bronchi and bronchioles were also present. Alveolar septa were expanded by edema fluid and leucocytes. Moderate type II pneumocyte hyperplasia was noted in more consolidated areas with abundant alveolar macrophages, cellular exudate and edema. Blood vessels were surrounded by moderate numbers of lymphocytes that multifocally aggregated in vascular tunics and elevated the overlying epithelium. Low numbers of syncytial cells were noted in the bronchioles and alveoli. These described lesions affected between 20–50% of pulmonary tissue in the vehicle control groups and while the pre-infection and post-infection treatment groups had similar lesions, they were significantly less abundant. One animal in each of the pre- and post-infection treatment groups had no lesions at all. Pneumonia in the remaining animals affected roughly 5–15% of the lung tissue, but lesions were minimal to mild.

**Figure 4:**
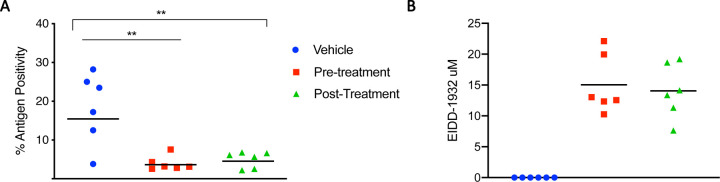
Morphometric analysis of viral antigen and drug concentration in the lungs. (**A**) A longitudinal cross section of the right lung was stained for viral antigen and scanned to measure the total amount of viral antigen present in the lung section. (**B**) EIDD-1931 concentrations in the lungs. (**A and B**) Blue circle, vehicle control; red square, pre-infection treatment; green triangle, post-infection treatment. **Summary of results:** (**A**) The area of lung staining positive for viral antigen showed a statistically significant difference between both of the MK-4482 treatment groups, compared to vehicle controls. No difference between individual treatment groups was present. For A and B, means are shown. ANOVA followed by Kruskal-Wallis analysis and a pairwise Wilcox test was used to analyze differences among groups. **p<0.008

Immunoreactivity against SARS-COV2 antigen was used to further compare the lung samples between the three different treatment groups (Figure 3G to I). Antigen staining was observed in bronchial and bronchiolar epithelium, type I and II pneumocytes as well as a small number of pulmonary macrophages. A positive pixel analysis on whole lung slides demonstrated a significant difference in viral antigen present among the three groups. The total number of positive pixels was divided by the area of lung scanned to determine a percentage of lung containing viral antigen. This analysis revealed that the vehicle controls contained significantly more antigen than the treated groups, with the vehicle controls having on average 4.71 times more antigen signal than pre-infection treatment animals and 3.68 times more signal than post-infection treatment animals. Post-infection treatment animals exhibited a slightly higher antigen signal than pre-infection treatment animals, but the difference was not significant (Figure 4A).

To evaluate the pharmacokinetics of MK-4482 in the animals, MK-4482 and the EIDD-1931 metabolite were measured in clarified lung homogenate by liquid chromatography and mass spectrometry (LCMS) at the point of necropsy. Since SARS-CoV-2 is a respiratory disease, levels of drug in lung tissue are expected to be the best indicator of therapeutic potential. All treated animals displayed detectable levels of EIDD-1931 in the lung and levels were similar across treatment groups (pre-infection: 18.80 ± 5.97 nmol/g_lung_, post-infection 17.56 ± 5.49 nmol/g_lung_) (Table 1) (Figure 4B). In line with its demonstrated rapid hydrolysis to EIDD-1931 following absorption, MK-4482 was not detected in the tissue^12,15^. Volume/concentration is difficult to estimate in tissues due to non-homogenous drug distribution and organ hydration. On average, water content of the lung is approximately 80% by weight and this number can be used to calculate a conservative estimated EIDD-1931 concentration in the tissue under the assumptions of homogenous distribution and hydration^18^. These estimates suggest a concentration of 15.04 ± 4.78 μM in the pre-infection group and 14.05 ± 4.39 μM in the post-infection group at the point of necropsy (12 hours post final MK-4482 dose) (Table 1) (Figure 4B). These values compare well with previous studies where a single oral dose of MK-4482 at 128mg/kg in ferrets (compared to 250mg/kg in our study) resulted in EIDD-1931 lung concentrations of 10.7 ± 1.2nmol/g^12^.

## DISCUSSION

In the present study, we used the established Syrian hamster animal model^16,17^ to assess the inhibitory effect of the nucleoside analog MK-4482 on SARS-CoV-2 replication *in vivo*. Our study shows the capacity of MK-4482 to substantially reduce the replication of SARS-CoV-2 in the lungs based on both viral RNA genome copy number and levels of infectious virus. Importantly, this control of virus replication was associated with markedly reduced lung pathology. MK-4482 has been shown to inhibit the replication of other related human coronaviruses, MERS-CoV and SARS-CoV-1 in mouse models^15^. Our current study is the first demonstration of inhibition of SARS-CoV-2 and amelioration of lung disease by MK-4482 in any animal model.

Currently, only a single drug (GS-5734) has been given EUA for treatment of SARS-CoV-2 induced COVID-19 disease^9^. Rather than having an impact on mortality, the EUA was based on a demonstration of reduced recovery time for hospitalized patients with COVID-19^10^. In a study performed in the rhesus macaque model, GS-5734 administered at 12 hours post-infection was shown to lower both the peak infectious titers of SARS-CoV-2 in bronchoalveolar lavage (BAL) and virus genome copy number in the lung at day 7 post-infection by approximately 2-logs^11^. Currently, there is no data showing the efficacy of GS-5734 against SARS-CoV-2 in the Syrian hamster model, but treatment starting a day prior to infection and continued twice daily thereafter resulted in significant improvement of SARS-CoV-2 infection in adenovirus 5-hACE2 transduced mice^19^. However, the hamster and macaque models appear relatively comparable, with both being associated with a rapid increase in SARS-CoV-2 replication in the lung and other respiratory tissues and mild clinical disease^16,17,20^. Given these similarities, MK-4482 should likely be considered as a potential additional treatment option for COVID-19 patients.

Similar to GS-5734, MK-4482 exhibits broad inhibition of divergent RNA viruses^12–15,21–23^. Although both drugs are nucleoside analogs, MK-4482 has been shown to function as a RNA mutagen inducing genome catastrophe^15,24^, while GS-5734 is a non-obligate RNA chain terminator^25^. The function of MK-4482 as an RNA mutagen may raise concerns regarding ‘off-target’ mutagenic toxicity. However, even at an EIDD-1931 dose of 500mg/kg, treatment of mice in a MERS-CoV model did not increase mutation rates of the ISG15 mRNA transcript, a gene highly induced during MERS-CoV infection, whilst viral genes accumulated mutations^15^. Incorporation of ribonucleosides has also been shown to be highly selective for RNA compared to host DNA^26^. Consistent with this level of safety, the guanosine ribonucleoside analog, ribavirin, which has several mechanisms of action including one of RNA mutation/error catastrophe, has been used for decades in patients, including infants with severe lower respiratory tract infections^27^. If deemed safe, MK-4482 would join GS-5734 as the second broadly direct acting antiviral to target emerging RNA viruses, and in this case, specifically SARS-CoV-2.

Infectious disease pathology is a complex interplay between the pathogen and the host. Consequently, strategically planned combination therapy may be more effective than the use of single drugs. Combinations of drugs with different mechanisms of action would be preferable. Such combination therapy has been shown to be highly effective for the control of other viral pathogens, notably human immunodeficiency and hepatitis C virus infection^28,29^. Therefore, the combination of MK-4482, an RNA mutagen, with the non-obligate RNA chain terminator, GS-5734, may yield additional efficacy in the treatment of SARS-CoV-2 infections. Additional combination partners could be potent neutralizing antibodies^30^. In addition, immune response modifying drugs such as dexamethasone have been shown to be effective for the later deleterious host responses associated with COVID-19 disease^31^. The combination of such a therapeutic with direct antivirals, such as MK-4482 and GS-5734, may increase treatment efficacy, especially in more severe cases.

GS-5734 is currently only administered by the intravenous route. A clear advantage of MK-4482 is the capacity for oral administration, which opens up the possibility for use of the drug as a post-exposure treatment prior to symptom onset. Our data suggest that initiation of treatment within 12 hours of a productive exposure resulting in infection significantly reduces SARS-CoV-2 replication and associated pathology in the lung target organ. Consistent with this idea, direct acting antivirals, including GS-5734 have been shown to be most effective in modifying disease outcome when administered early following infection^32^. If adequately priced for widespread global use, we believe this post-exposure application MK-4482 could substantially affect the course of the pandemic.

## MATERIALS AND METHODS

### Biosafety and ethics

Work with infectious SARS-CoV-2 was approved by the Institutional Biosafety Committee (IBC) and performed in high biocontainment at Rocky Mountain Laboratories (RML), NIAID, NIH. Sample removal from high biocontainment followed IBC-approved Standard Operating Protocols. Animal work was approved by the Institutional Animal Care and Use Committee and performed by certified staff in an Association for Assessment and Accreditation of Laboratory Animal Care International accredited facility. Work followed the institution’s guidelines for animal use, the guidelines and basic principles in the NIH Guide for the Care and Use of Laboratory Animals, the Animal Welfare Act, United States Department of Agriculture and the United States Public Health Service Policy on Humane Care and Use of Laboratory Animals. Syrian hamsters were group housed in HEPA-filtered cage systems enriched with nesting material and were provided with commercial chow and water *ad libitum*. Animals were monitored at least twice daily.

### Virus and cells

SARS-CoV-2 isolate nCoV-WA1–2020 (MN985325.1) was kindly provided by the Centers for Disease Control and Prevention, Atlanta, GA, USA^33^ and propagated once at RML in Vero E6 cells in high glucose DMEM (Sigma) supplemented with 2% fetal bovine serum (Gibco), 1 mM L-glutamine (Gibco), 50 U/ml penicillin and 50 μg/ml streptomycin (Gibco). The virus stock used was free of contaminations and confirmed to be identical to the initial deposited Genbank sequence (MN985325.1). Vero E6 cells were maintained in high glucose DMEM supplemented with 10% fetal calf serum, 1 mM L-glutamine, 50 U/mL penicillin and 50 μg/mL streptomycin.

### Syrian hamster study design

Hamsters were divided into groups for either pre-infection or post-infection MK-4482 treatment (n=6 per group). Groups were then treated with MK-4482 (250 mg/kg) [MedChemExpress dissolved in 10 % polyethylene glycol (PEG)-400; 2.5% Cremophor RH40 in water] at 12 hours and 2 hours prior to infection (pre-infection group) or 12 hours following infection (post-infection group). Treatment was then maintained with 12 hour dosing until the completion of the study 84 hours post-infection (day 4). A third group consisted of vehicle control animals that received the same dosing schedule and volume as the pre-infection group. All groups were infected intranasally with 5×10^2^ TCID_50_ of SARS-CoV-2 (25 μL/nare). Animals were monitored twice daily for disease signs and progression. All procedures were performed on anesthetized animals. Oral swabs were collected on days 2 and 4 post-infection. Animals were euthanized on day 4-post infection and lung tissues were collected at necropsy for pathology and virology.

### Liquid chromatography and mass spectrometry (LCMS)

LCMS grade water, methanol, acetonitrile and acetic acid were purchased through Fisher Scientific. All synthetic standards for molecular analysis were purchased from MedChemExpress. Clarified lung homogenates were gamma-irradiated (2 megarads) for removal from biocontainment according to IBC-approved protocol^34^. Standard curves of MK-4482 and EIDD-1931 were made in lung homogenate from uninfected animals and subjected to irradiation to account for molecular degradation. Samples were prepared for analysis by adding 300 μL of methanol to 100 μL of homogenate and incubating at 4°C for 30 minutes to precipitate macromolecules. Samples were centrifuged at 16,000 × g at 4°C and the supernatant was transferred to a sample vial for LCMS analysis. Samples were separated by HILIC chromatography on a Sciex ExionLC™ AC system. Samples were injected onto a Waters XBridge® Amide column (130Å, 3.5 μm, 3 mm × 100 mm) and eluted using a binary gradient from 95 % acetonitrile, 0.8 % acetic acid, 10 mM ammonium acetate to 50 % acetonitrile, 0.8 % acetic acid, 10 mM ammonium acetate over 8 min. Analytes were measured using a Sciex 5500 QTRAP® mass spectrometer in positive mode with electrospray ionization (CUR: 40, CAD: Med, ISV: 2500, Temp: 450, GS1: 50, GS2: 50). Multiple reaction monitoring (MRM) was performed using the optimized conditions in Table 2. To ensure signal fidelity triggered spectra were compared back to synthetic standards. Previously published MRM signals for biological nucleosides were utilized to confirm minimal interference at the retention time of interest^35^. All analytes were quantified against an 8-point calibration curve of the respective synthetic standard prepared in the target matrix and processed in the same manner as experimental samples. Limits of quantification in lung homogenate after irradiation was 5 ng/mL for EIDD-1931 and 50 pg/mL for MK-4482.

### Virus load

RNA was extracted from swabs using the QIAamp Viral RNA kit (Qiagen) according to the manufacturer’s instructions. Tissues were homogenized in RLT buffer and RNA was extracted using the RNeasy kit (Qiagen) according to the manufacturer’s instructions. For detection of viral RNA, 5 μl RNA was used in a one-step real-time RT-PCR against the N gene which detects genomic and subgenomic RNA^17^ using the Rotor-Gene probe kit (Qiagen) according to instructions of the manufacturer. In each run, standard dilutions of RNA standards counted by droplet digital PCR were run in parallel, to calculate copy numbers in the samples.

### Virus titration

Virus isolation was performed on lung tissues by homogenizing the tissue in 1 mL DMEM using a TissueLyser (Qiagen) and inoculating Vero E6 cells in a 96-well plate with 200 μL of a 1:10 dilution series of the cleared homogenate. One hour after inoculation of cells, the inoculum was removed and replaced with 200 μL DMEM (Sigma-Aldrich) supplemented with 2% fetal bovine serum, 1 mM L-glutamine, 50 U/mL penicillin and 50 μg/mL streptomycin. Six days after inoculation, cytopathogenic effect was scored and the TCID_50_ was calculated using the Reed-Muench method^36^.

### Histopathology and immunohistochemistry

Histopathology and immunohistochemistry were performed on hamster lung tissues. Tissues were fixed in 10 % Neutral Buffered Formalin with two changes, for a minimum of 7 days according to IBC-approved SOP. Tissues were placed in cassettes and processed with a Sakura VIP-6 Tissue Tek, on a 12-hour automated schedule, using a graded series of ethanol, xylene, and PureAffin. Embedded tissues were sectioned at 5 μm and dried overnight at 42°C prior to staining. Specific anti-CoV immunoreactivity was detected using Sino Biological Inc. SARS-CoV/SARS-CoV-2 nucleocapsid antibody (Sino Biological cat#40143-MM05) at a 1:1000 dilution. The secondary antibody was the Vector Laboratories ImPress VR anti-mouse IgG polymer (cat# MP-7422). The tissues were then processed for immunohistochemistry using the Discovery Ultra automated stainer (Ventana Medical Systems) with a ChromoMap DAB kit (Roche Tissue Diagnostics cat#760–159). The tissues slides were scanned with the Aperio ScanScope XT (Aperio Technologies, Inc.) and the entire section analyzed with the ImageScope Positive Pixel Count algorithm (version 9.1)^37^. All tissue slides were analyzed by a board-certified veterinary pathologist.

### Statistical analyses

Statistical analysis was performed in R version 4.0.2. The difference in viral load, infectious titers and antigen positivity between study arms was assessed by ANOVA followed by a Kruskal-Wallis test and a pairwise Wilcoxon rank sum test to correct for multiple comparisons.

### Data Availability

All raw data (RT-PCR, infectious titers, pathology) is available upon reasonable request.

## Figures and Tables

**Table 1:** Lung concentrations of EIDD-1931

	EIDD-1931 Lung Concentration						
	Vehicle control						Avg ± Std
nmol/gram	0	0	0	0	0	0	0
Estimated concentration μM	0	0	0	0	0	0	0
	Pre-treatment						Avg ± Std
nmol/gram	16.29	15.43	27.62	12.82	24.93	15.69	18.80 ± 5.97
Estimated concentration μM	13.03	12.34	22.1	10.25	19.95	12.55	15.04 ± 4.78
	Post-treatment						Avg + Std
nmol/gram	23.98	9.54	23.3	17.67	14.15	16.72	17.56 ± 5.49
Estimated concentration μM	19.18	7.63	18.64	14.14	11.32	13.37	14.05 ± 4.39

**Table 2:** LCMS/MS MRM source conditions for the quantification of MK-4482 and EIDD-1931 **Key:** MRM: multiple reaction monitoring; DP: declustering potential; EP: entrance potential; CE: collision cell entrance potential; CXP: collision cell exit potential

Molecule	MRM pair	DP(V)	EP(V)	CE(V)	CXP (V)
MK-4482	330.0/128.0*	70	10	20	15
MK-4482	330.0/110.0	60	10	40	15
EIDD-1931	260.0/128.0*	90	10	20	15
EIDD-1931	260.0/110.0	110	10	50	15

* Signal used for quantification
